# Atypical basic movement kinematics in autism spectrum conditions

**DOI:** 10.1093/brain/awt208

**Published:** 2013-08-26

**Authors:** Jennifer L. Cook, Sarah-Jayne Blakemore, Clare Press

**Affiliations:** 1 UCL Institute of Cognitive Neuroscience, 17 Queen Square, London, WC1N 3AR, UK; 2 Donders Centre for Cognitive Neuroimaging, Radboud University, P.O. Box 9101, 6500 HB Nijmegen, The Netherlands; 3 Department of Psychological Sciences, Birkbeck, University of London, London, WC1E 7HX, UK

**Keywords:** autism, kinematics, biological motion, motor control

## Abstract

Individuals with autism spectrum conditions have difficulties in understanding and responding appropriately to others. Additionally, they demonstrate impaired perception of biological motion and problems with motor control. Here we investigated whether individuals with autism move with an atypical kinematic profile, which might help to explain perceptual and motor impairments, and in principle may contribute to some of their higher level social problems. We recorded trajectory, velocity, acceleration and jerk while adult participants with autism and a matched control group conducted horizontal sinusoidal arm movements. Additionally, participants with autism took part in a biological motion perception task in which they classified observed movements as ‘natural’ or ‘unnatural’. Results show that individuals with autism moved with atypical kinematics; they did not minimize jerk to the same extent as the matched typical control group, and moved with greater acceleration and velocity. The degree to which kinematics were atypical was correlated with a bias towards perceiving biological motion as ‘unnatural’ and with the severity of autism symptoms as measured by the Autism Diagnostic Observation Schedule. We suggest that fundamental differences in movement kinematics in autism might help to explain their problems with motor control. Additionally, developmental experience of their own atypical kinematic profiles may lead to disrupted perception of others’ actions.

## Introduction

Studies of motor control suggest that complex movements, once decomposed, consist of common signatures; thus by studying simple movements it is possible to gain insight into the more complex sequences that comprise ‘actions’ (Yazdani *et al.*, 2012). To this end considerable effort has been invested in characterizing the fundamental kinematics that underpin simple movements that proceed from one point in space to another. Such point-to-point movements follow a bell-shaped velocity profile that can be described by the minimum jerk and two-thirds power law equations (Flash and Hogan, 1985; Todorov and Jordan, 1998). These equations formalize the observation that humans produce smooth actions. For example, when reaching out to grasp a glass, a person will initially accelerate steadily then, as they approach their goal, gradually decrease the velocity of their arm movement. Such laws of motion comprise a general organizing principle that underpins both gross movements such as sinusoidal arm waving (Abend *et al.*, 1982; Flash and Hogan, 1985; Todorov and Jordan, 1998) and more intricate movements including drawing (Viviani and Terzuolo, 1982) and handwriting (Edelman and Flash, 1987). Humans use a model of this bell-shaped velocity profile to predict the actions of others (Stadler *et al.*, 2012), and are better at perceiving deviations from it than from other familiar velocity profiles, such as gravitational motion (Cook *et al.*, 2009).

Individuals with an autism spectrum condition (autism hereafter) are characterized by repetitive and stereotyped interests, and difficulties with social interaction and communication (American Psychiatric Association, 1994). In addition to these core features children and adults with autism exhibit motor difficulties. For example, individuals with autism exhibit difficulties controlling the force and direction of a ball when throwing (Staples and Reid, 2010) and differ from typical individuals with respect to handwriting (Beversdorf *et al.*, 2001). Furthermore, when executing motor tasks, they demonstrate atypical activation in the cerebellum and supplementary motor area, as well as reduced connectivity between motor nodes (Mostofsky *et al.*, 2009). Motor difficulties in autism can be identified at both the level of gross and fine motor control (Beversdorf *et al.*, 2001; Mostofsky *et al.*, 2006; Gowen and Hamilton, 2013), suggesting a possible underlying problem with fundamental movement kinematics.

A parallel line of research suggests atypicalities in autism in the visual perception of human movement. These perceptual difficulties have been identified both with whole body stimuli (Blake *et al.*, 2003; Klin *et al.*, 2009; Annaz *et al.*, 2010, 2012; Kaiser *et al.*, 2010*a*; but see Murphy *et al.*, 2009; Saygin *et al.*, 2010; Jones *et al.*, 2011), and with stimuli that comprise a single hand executing sinusoidal movements requiring sensitivity to the bell-shaped velocity profile (Cook *et al.*, 2009).

At least two putative mechanistic pathways link the visual perception and the execution of action. First, the motor system may contribute directly to perception. This hypothesis has received much attention over the last decade due to the discovery of mirror neurons, which respond to both action observation and execution. Such neurons have been found in the ventral premotor cortex (area F5; di Pellegrino *et al.*, 1992; Gallese *et al.*, 1996) and inferior parietal lobule (Fogassi *et al.*, 2005) of the macaque, and homologous regions exhibit similar response properties in the human brain (Iacoboni *et al.*, 1999; Grèzes *et al.*, 2003; Chong *et al.*, 2008; Gazzola and Keysers, 2009; Kilner *et al.*, 2009). Supporting the hypothesis that the motor system contributes to perception, studies have demonstrated that employing transcranial magnetic stimulation to disrupt activity in classic mirror neuron regions, such as the premotor cortex, decreases sensitivity to the perception of whole-body biological motion (van Kemenade *et al.*, 2012), and that one’s own motor experience can enhance perception of others’ actions (Aglioti *et al.*, 2008). The second putative mechanistic pathway concerns the role that action execution may play in the development of the visual system. Infants spend a large proportion of their time during early development watching their own limb movements (White *et al.*, 1964; van der Meer *et al.*, 1995; Rochat, 1998), which follow the two-thirds power law (Hofsten and Rönnqvist, 1993). Given that early visual experiences play an important role in tuning the visual system (Blakemore and Cooper, 1970; Sangrigoli *et al.*, 2005) an individual’s early visual experiences of their own kinematics may have a significant impact on their sensitivity to the kinematics of others’ actions.

Therefore, at least two lines of research suggest potential atypical kinematics in autism. First, individuals with autism exhibit difficulties with both gross and fine motor control. Second, individuals with autism differ from typical individuals in their visual perception of biological kinematics; thus, given that action execution and perception are linked, they may also differ from controls with respect to the kinematics of movement execution.

The present study used motion tracking technology to record kinematics (velocity, acceleration and jerk) while adults with autism and a matched typical control group performed simple sinusoidal arm movements. It was hypothesized that adults with autism would differ from control individuals in terms of the basic kinematics of movements. In addition, to investigate whether, in our sample of individuals with autism, execution of kinematics is linked to perception, we correlated performance on this task with that in a secondary biological motion perception task.

## Materials and methods

### Primary task

#### Participant details

Participants were recruited from the University College London (UCL) subject pool and the UCL Institute of Cognitive Neuroscience Autism database. All participants had normal or corrected-to-normal vision and were screened for exclusion criteria (dyslexia, epilepsy, and any other neurological or psychiatric conditions). Fifteen control participants and 14 participants with autism were recruited. Participants in the autism group had a diagnosis of autism, Asperger’s syndrome or autism spectrum disorder from an independent clinician. The Autism Diagnostic Observation Schedule (ADOS; Lord *et al.*, 1989) was administered by a researcher trained and experienced in the use of this semi-structured observation schedule. All participants met cut-off for a diagnosis of autism spectrum on the total ADOS score [mean standard error of the mean (SEM) = 10.36 (0.84); cut-off = 7] and on the communication [mean (SEM) = 3.50 (0.27); cut-off = 2] and reciprocal social interaction [mean (SEM) = 6.93 (0.66); cut-off = 4] subscales. Groups were matched for age [autism mean (SEM) = 41.07 (3.80) years; control = 37.60 (3.89); *t*(27) = −0.64, *P* = 0.53], gender (autism M:F = 11:3; control M:F = 13:2; **χ^2^** = 0.01, *P* = 0.92) and full scale IQ as measured by the Wechsler Abbreviated Scale of Intelligence (Wechsler, 1999) [autism mean (SEM) = 114.36 (3.56); control = 118.93 (2.30); *t*(27) = 1.09, *P* = 0.28]. All participants gave informed consent to take part in the study, which was approved by the local ethics committee and performed in accordance with the ethical standards laid down in the 1964 Declaration of Helsinki.

#### Procedure

An infrared-based Vicon motion tracking system (http://www.vicon.com/) was employed to record kinematics while participants completed a movement execution task wherein they conducted simple horizontal sinusoidal (back and forth) right arm movements. Infrared reflective markers on participants’ arms were monitored by six cameras during execution. The *x*, *y* and *z* plane coordinates from the right finger were recorded at a rate of 100 Hz. The first six movements were accompanied by an auditory tone, which encouraged all participants to move at approximately the same rate. Participants completed five blocks of 10 movements. Data were collected as part of a larger test battery which, for the individuals with autism, included the biological motion perception task.

#### Data analysis

Velocity for each movement was calculated as the square root of the sum of the squared differentials of the *x*, *y* and *z* vectors. Vectors were low pass filtered at 10 Hz, and 10 data points were trimmed from the end of each vector to remove artefacts associated with the filter. Acceleration and jerk were calculated as the first and second order differentials of these vectors. Distance travelled was estimated by multiplying the mean velocity vector by the number of datapoints for each participant. Independent sample *t*-tests were employed to compare means. All *t*-tests reported are two-tailed.

To analyse the time course of velocity, acceleration and jerk, independent of size of movement vector, movement vectors were resampled and trimmed such that the velocity profile was maintained but vectors were equated in length (all vectors comprised 106 data points). Individual participant resampled vectors were filtered, trimmed and differentiated in the same manner as the analyses of raw vectors.

Time-course analyses were conducted using a previously implemented method (Press *et al.*, 2011). Forty-eight data points at the ends of the vectors (24 at each end) were compared with 48 data points from the middle. ANOVAs (2 × 2) were conducted with a between-subjects factor group (autism vesus control) and a within-subjects factor timepoint (end versus middle).

Given the high correlation between velocity, acceleration and jerk, we sought to obtain a single score that characterized the kinematics of an individual’s movements. This was achieved by performing factor analysis on velocity, acceleration and jerk scores, using the regression method. To investigate whether there was an association between kinematics and autism severity, the resulting kinematics factor scores were correlated with ADOS total scores (Lord *et al.*, 1989).

### Perception task

#### Participant details

All but one of the participants with autism that took part in the primary experiment also took part in the perception task. Control participants did not take part in the perception task.

#### Procedure

**Figure 1:**
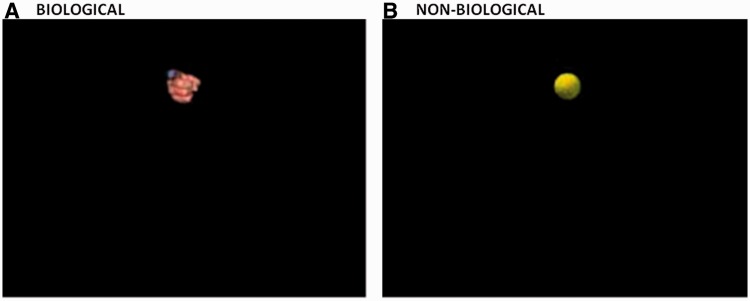
In the perception task, participants watched a single animation that showed a biological stimulus (**A**; a hand) or a non-biological stimulus (**B**; a tennis ball) moving vertically across the screen. In each trial, the velocity profile of the movement was either 100% natural motion (minimum jerk in the biological condition; gravitational in the non-biological condition), or 100% constant velocity or some linear combination of the two extremes. In each trial the task was to judge whether the stimulus moved in a ‘natural’ or ‘unnatural’ way.

Participants watched a series of visual stimuli constituting two conditions: biological (minimum jerk) motion and non-biological (gravitational) motion. For the biological condition an image of a human hand (Fig. 1A) was programmed to make a vertical sinusoidal movement (down and then up) of amplitude 110 mm and frequency 0.5 Hz. The velocity profile of the stimulus was generated by motion-morphing between two movement prototypes. Prototype 1 was described by a constrained minimum jerk model (Todorov and Jordan, 1998), and Prototype 2 was described by a constant velocity vector. For the non-biological condition an image of a tennis ball (Fig. 1B) was programmed to make a vertical downward movement of amplitude 215 mm and frequency of 1 Hz. Thus, the tennis ball appeared from the top of the screen and disappeared off the bottom of the screen. The velocity profile was generated by motion-morphing between two prototypes: Prototype 1 was described by the standard equation of gravitational motion *h*(*t*) = *h0* – 0.5 *gt*^2^, where *h* = height, *h0* = initial height, *t* = time and *g* = gravitational force (9.8 m/s^2^); and Prototype 2 was a constant velocity vector. Motion morphing adhered to the following equation:

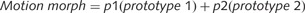

where the weights p*i* determine the proportion of the morph described by the individual prototype. There were 26 motion-morph levels for each condition, spanning 100% constant velocity to 0% constant velocity in 4% steps. Six animations per motion-morph level were shown, meaning that participants watched 156 animations per condition, and 312 animations in total.

In each trial participants watched a single animation. The task was to indicate whether the stimulus moved in a ‘natural’ or ‘unnatural’ way (Supplementary material). Participants could take as long as they wanted to make their decision. Participants completed six blocks of 52 trials—three biological blocks and three non-biological blocks. Within a block, trials were pseudo-randomized such that any one motion-morph level was never presented on three consecutive trials. Block order within participants was also pseudo-randomized according to the three consecutive rule. New motion-morph and block order randomizations were generated for each participant. The duration of the entire experiment including breaks was ∼45 min.

#### Data analysis

Data were modelled by fitting cumulative Gaussians to estimate psychometric functions. Function fitting was completed in MATLAB using the Palamedes toolbox (Kingdom and Prins, 2009; Supplementary material). Separate functions for biological and non-biological tasks were modelled for each participant, and the point of subjective equivalence was estimated. The point of subjective equivalence denotes the ratio of ‘signal’ (minimum jerk or gravitational) to noise (constant velocity) at the point where participants are equally likely to judge a stimulus as natural or unnatural. Thus a high point of subjective equivalence indicates a bias towards unnatural judgements even when the stimulus comprises a high proportion of objectively natural motion. For example, a high point of subjective equivalence in the biological condition would indicate that, despite the fact that the stimulus comprises a high ratio of minimum jerk to constant velocity, the participant judges it as natural only 50% of the time, thus demonstrating a bias towards unnatural judgements. The points of subjective equivalence from the biological and non-biological tasks were correlated with the kinematics factor score derived from the primary task. All reported *P*-values are two-tailed.

## Results

### Primary task

**Figure 2:**
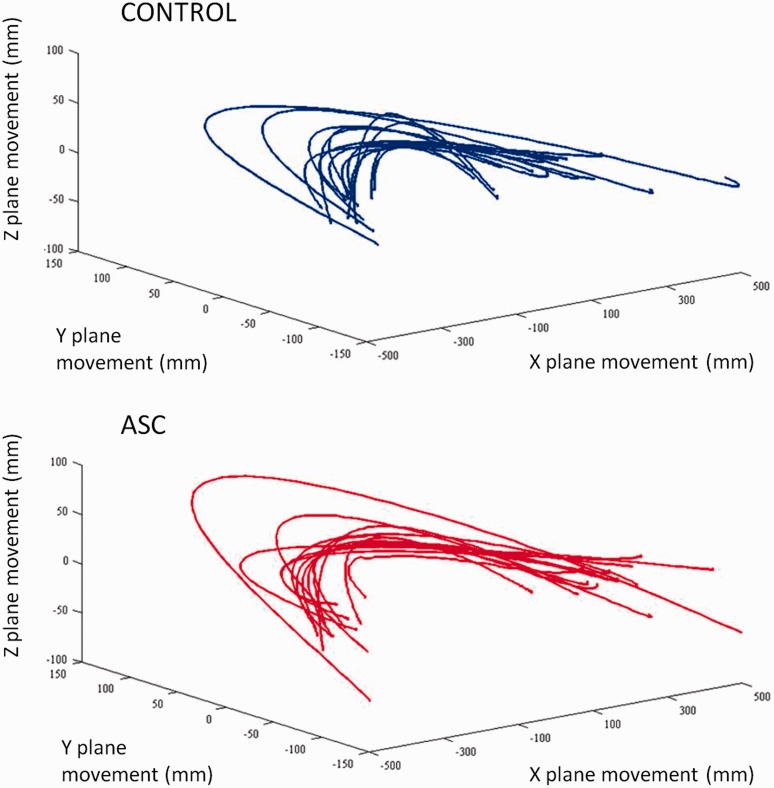
Mean corrected *x*, *y* and *z* coordinates for movements conducted by controls (blue) and individuals with autism (red) in the primary task. Individuals with autism and control participants executed movements that followed similar paths through space.

**Figure 3:**
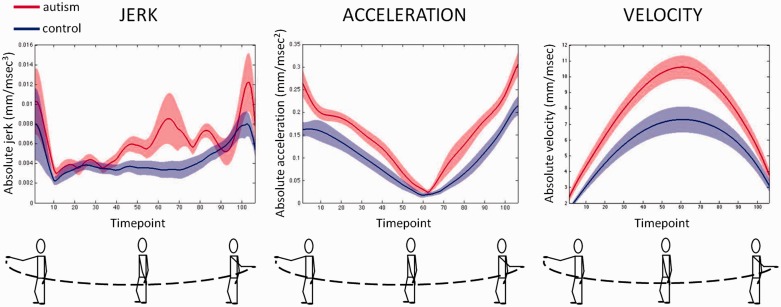
Basic kinematics of arm movements for control participants and individuals with autism in the primary task. When executing simple sinusoidal arm movements individuals with autism made more jerky movements (*left*) and travelled with faster absolute acceleration (*middle*) and velocity (*right*). Mean movement vectors are plotted in red for the autism group and blue for the control group. Shaded regions indicate the standard error of the mean.

Participants completed 50 left-to-right or right-to-left arm movements (with the exception of two participants with autism, who completed 40 and 30 movements, respectively). Despite following similar 3D paths through space (Fig. 2), the kinematics of the movements conducted by individuals with autism differed significantly from those conducted by control participants (Fig. 3). Participants with autism produced more ‘jerky’ movements than typical adults [mean (SEM) absolute jerk (mm/ms^3^) for the autism group: 0.026 (0.0030); control group: 0.015 (0.0016); *t*(27) = −3.28, *P* = 0.003, Cohen’s *d* = −1.26] and moved with greater absolute acceleration [mean (SEM) in mm/ms^2^ for the autism group: 0.33 (0.030); control: 0.19 (0.023); *t*(27) = −3.75, *P* = 0.001, Cohen’s *d* = −1.44]. On average the autism group also moved for a shorter duration [mean (SEM) in ms for the autism group: 810.02 (10.77); control: 844.07 (10.96); *t*(27) = 2.21, *P* = 0.04, Cohen’s *d* = 0.85] and travelled further [mean (SEM) in mm for the autism group: 1075.77 (75.76); control: 745.14 (78.96), *t*(27) = −3.01, *P* = 0.006, Cohen’s *d* = −1.16]. Hence the autism group exhibited faster absolute velocity [mean (SEM) in mm/ms for the autism group: 13.33 (1.01); control: 8.84 (0.94), *t*(27) = −3.27, *P* = 0.003, Cohen’s *d* = −1.26]. For all analyses the pattern of significance remained irrespective of whether squared or absolute values were analysed.

To investigate whether differences between the groups in kinematics were independent from differences in movement distance, we performed an additional analysis comparing the autism group with a subset of the control group matched on this dimension. Controls were ranked according to average movement distance and one-third were removed to leave a subgroup of 10 control participants who did not differ from the autism group in terms of distance (nor in terms of age, IQ, or velocity, all *P* > 0.05). This subgroup of control participants significantly differed from the autism group with respect to acceleration [*t*(23) = 2.37, *P* < 0.05, Cohen’s *d* = −1.01] and jerk [*t*(23) = 2.12, *P* < 0.05, Cohen’s *d* = −0.92]. Thus the differences between the groups in acceleration and jerk remain when accounting for the distance of executed movements.

Figure 3 illustrates how velocity, acceleration and jerk changed as a function of time for the two groups of participants. Comparing the endpoints of movements with midpoints revealed an interaction between group and timepoint in velocity. This interaction reflected the greater increase in velocity from end- to mid-points for the autism group compared with the control group [*F*(1,27) = 7.98, *P* = 0.01, η_p_^2^ = 0.23]. There was no interaction between group and timepoint for jerk or acceleration (all *F* < 2.96, all *P* > 0.10), indicating that jerk and acceleration effects were independent of movement phase (see Supplementary Table 1 for all significant main effects and interactions).

**Figure 4:**
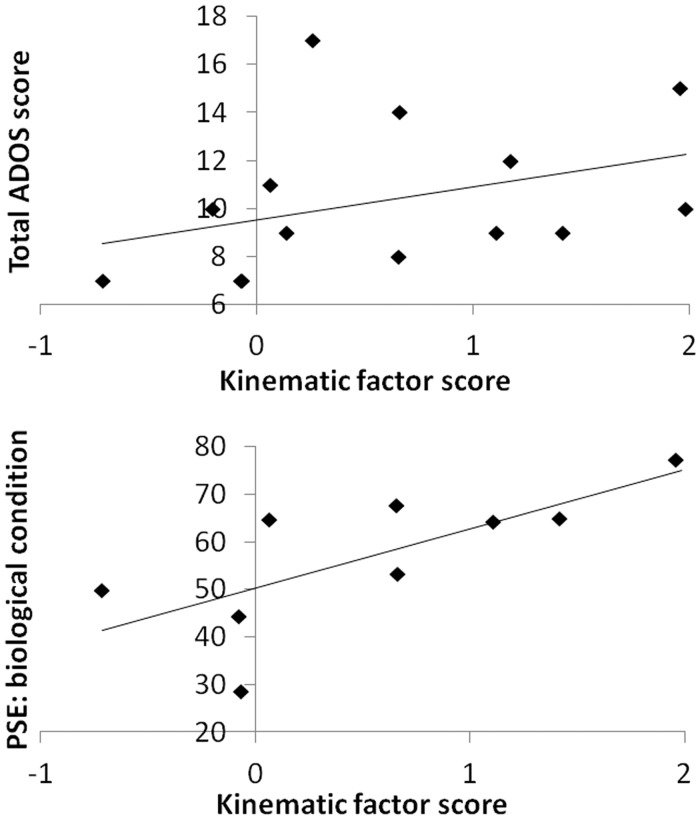
Correlations between kinematics and ADOS score, and kinematics and biological motion perceptual categorization. *Top*: Scattergraph with regression line depicting the relationship between the kinematic factor score and total ADOS score. The magnitude of kinematic atypicality was positively correlated with autism severity as measured by the ADOS (Lord *et al.*, 1989). *Bottom*: Similar scattergraph depicting the relationship between the kinematic factor score and point of subjective equivalence (PSE) in the biological condition of the categorization task. The magnitude of kinematic atypicality was positively correlated with bias towards unnatural categorization.

Kinematic factor scores and ADOS total scores (Lord *et al.*, 1989) were significantly positively correlated (Spearman’s rho = 0.56, *P* = 0.04; Fig 4). Thus, individuals who produced more atypical kinematics exhibited greater autism symptom severity.

### Perception task

Two participants did not generate adequate data for psychometric function fitting and three participants generated suitable data in only one condition. Thus the reported statistics are based on 11 data sets for the biological condition and 10 data sets for the non-biological condition.

Points of subjective equivalence for the biological task, but not for the non-biological task, were positively correlated with the kinematic factor score (biological condition: Spearman’s rho = 0.61, *P* = 0.04, Fig 4; non-biological condition: Spearman’s rho = −0.20, *P* = 0.58). The pattern of significance was the same if only the data sets for the nine participants who generated data suitable for psychometric fitting for both conditions were used (biological condition: Spearman’s rho = 0.75, *P* = 0.02; non-biological condition: Spearman’s rho = 0.10, *P* = 0.80).

## Discussion

Individuals with autism produced horizontal sinusoidal arm movements that were more jerky than those of control participants, and which proceeded with greater acceleration and velocity (Fig. 3). The magnitude of these atypicalities was significantly correlated with autism severity, as measured by the ADOS (Lord *et al.*, 1989), and with biased perception of biological motion (Fig. 4).

Given the importance of kinematics in both gross and fine motor control, atypicalities in this domain could be one reason for the difficulties with everyday motor control commonly experienced by individuals with autism (Beversdorf *et al.*, 2001; Gowen and Hamilton, 2013). A lack of typical kinematics might be a consequence of peripheral factors (Todorov, 2004) such as abnormal muscle tone in autism (Maurer and Damasio, 1982), or central nervous system (CNS) factors. One putative CNS factor is poor anticipation of the subsequent part of a motor sequence (Cattaneo *et al.*, 2007; Fabbri-Destro *et al.*, 2009). For instance, one study examined the time taken to reach for an object when it was to be subsequently placed on a large (easy condition) or small (difficult condition) target (Fabbri-Destro *et al.*, 2009). Control participants exhibited the typical pattern of a slower reach phase when the subsequent placing phase was more difficult, but the reaching movements of children with autism were not modulated by task difficulty. The authors concluded that, instead of translating their goal into a chain of motor acts, children with autism executed these acts independently. One possible explanation for the current results is that individuals with autism have a compromised ability to predict the point at which they must change the direction of their movement or difficulties with using this prediction to modulate current action kinematics. Another, potentially related, putative CNS factor that may contribute to atypical kinematics in autism is cerebellar neuropathology (Rogers *et al.*, 2013). Autism has been associated with cerebellar abnormalities including reduced Purkinje cell numbers (Courchesne *et al.*, 1988; Bauman, 1991; Courchesne, 1997; Palmen *et al.*, 2004; DiCicco-Bloom *et al.*, 2006), lower cerebellar vermal volumes (Webb *et al.*, 2009), reductions in the size and number of cells in the cerebellar nuclei, excess Bergmann glia and active neuroinflammatory processes within cerebellar white matter (Bailey *et al.*, 1998; Bauman and Kemper, 2005; Vargas *et al.*, 2005). A number of accounts suggest that cerebellar atypicalities play a key role in the development of the cognitive and behavioural profile that characterizes autism (Gowen and Miall, 2007; Mostofsky *et al.*, 2009; Rogers *et al.*, 2013). Further studies are necessary to assess the contribution of peripheral and central factors and to investigate whether they have specific or general effects on velocity, acceleration and jerk. Additionally, future work might address whether the same kinematic atypicalities are evident in autism when movements are constrained; for example by specifying that participants must move slowly or must interact with objects. These investigations would build on the present findings which highlight that in unconstrained situations, individuals with autism move atypically.

Within the social domain, the kinematics of action provide a great deal of information about the internal states of others. Action kinematics differ depending on the social context of the movement (Georgiou *et al.*, 2007; Becchio *et al.*, 2008*a*, *b*; Sartori *et al.*, 2009); for example, different reach-to-grasp movements are executed depending on whether the grasped object is to be given to another individual or placed in a holder (Becchio *et al.*, 2008*b*). Furthermore, a typical observer can predict an actor’s confidence in their judgements from the kinematics of their movements (Patel *et al.*, 2012) and can easily detect emotions from simple motion cues (Hubert *et al.*, 2007). In contrast, individuals with autism show poor performance on such emotion detection tasks (Hubert *et al.*, 2007; Parron *et al.*, 2008). Indeed, problems with perceiving and categorizing biological motion in autism have been reported from the age of 2 years (Klin *et al.*, 2009; Annaz *et al.*, 2012) through to adulthood (Blake *et al.*, 2003; Cook *et al.*, 2009; Kaiser *et al.*, 2010*a*; Nackaerts *et al.*, 2012); and the neural response to biological motion differs between individuals with autism and control participants (Herrington *et al.*, 2007; Freitag *et al.*, 2008; Kaiser *et al.*, 2010*b*). Robust evidence that action execution and perception are linked was one motivation for our hypothesis that, in addition to difficulties in perceiving biological kinematics, individuals with autism might execute actions that proceed with different kinematics relative to controls. The present findings of atypical kinematics during movement execution in autism are consistent with this hypothesis. Furthermore, the extent to which the kinematic profile of movement differed from the norm correlated with the tendency to classify observed biological, but not non-biological, movements as unnatural. This demonstrates that, in our sample of individuals with autism, execution atypicalities are linked to perception. Such findings might reflect influences of the motor system on perception (Aglioti *et al.*, 2008; van Kemenade *et al.*, 2012). Additionally, individuals with autism might develop a visual system that is tuned to atypical representations of biological motion from observing their own actions, which do not accord with typical kinematics.

The ability to communicate effectively through gesture and facial expression is one component of the ADOS (Lord *et al.*, 1989) assessment. Thus it may be hypothesized that the correlation we report between kinematic atypicalities and total ADOS score is due to atypical execution of communicative actions during the ADOS assessment. An alternative hypothesis builds on the theory that early problems with biological motion perception cause a cascade of impairments across development, including deficits in recognizing and understanding others’ actions and associated mental states (Klin *et al.*, 2009; Kaiser and Pelphrey, 2012). It may be hypothesized that atypical movement execution is linked to problems with the perception of biological motion, which may in turn cause a negative developmental cascade resulting in the social and communication difficulties indexed by the ADOS total score. It has previously been proposed that due to their movement atypicalities, individuals with autism may struggle to ‘simulate’, or mirror, the movements of others, potentially leading to socio-cognitive problems such as an inability to take the perspective, or understand the motivations, of other individuals (Gallese, 2006; Gallese *et al.*, 2009, 2013).

In further exploring such hypotheses research might also question the specificity of our finding to autism. For example, children with attention deficit hyperactivity disorder (ADHD) exhibit both motor (Pitcher *et al.*, 2003) and social difficulties (Uekermann *et al.*, 2010). Additionally, some motor abnormalities exhibited by individuals with autism are similar to those observed in individuals with cerebellar ataxia (e.g. irregular gait; Hallett *et al.*, 1993; Ambrosia *et al.*, 1988), and recent work suggests atypicalities in social functions such as theory of mind (Garrard *et al.*, 2008) and social emotion processing (D’Agata *et al.*, 2011) in ataxic individuals. However, whether atypical movement kinematics of the nature described in the present study are a feature of ADHD and cerebellar-ataxia is presently unknown. Further work in this field should also examine the importance of the developmental timescale of atypicalities; that is, are late- and early-occurring kinematic atypicalities equally likely to be associated with social difficulties?

The current findings have methodological implications for autism research in multiple fields. Videos and animations of typical human actions have been employed in many studies demonstrating impairments in autism—from those investigating biological motion perception to those examining belief inference. An important question concerns whether these impairments are also found when using action stimuli that match the kinematics of movements generated by individuals with autism. Preliminary evidence suggests that at least in some fields, impairments are specific to observation of typical movements. For example, Oberman *et al.* (2008) found reduced sensorimotor activity when individuals with autism viewed typical human actions but not when they viewed videos of their own actions. We hypothesize that this is because their own actions proceed with atypical kinematics.

In addition to methodological implications, the current results raise new questions concerning therapeutic interventions for, and early diagnosis of, autism. Importantly, they emphasize the use of sensorimotor therapies for individuals with autism. The efficacy of such therapies can be hindered by a lack of understanding of the causes of sensory and motor problems (Baranek, 2002). Speculating about the results of the current study, it could be argued that if individuals with autism can be trained to move with typical kinematics this might improve their perception of, and interactions with, the social world. Additionally, existing therapeutic interventions might be improved by using stimuli (such as robots) that can be programmed to move with kinematics characteristic of individuals with autism. The efficacy of therapeutic interventions may also be improved with earlier diagnosis of autism. It is argued that laws of motion such as the minimum jerk and two-thirds power law govern movement kinematics from birth. For instance, Hofsten and Rönnqvist (1993) found that, like those of adults, unconstrained non-goal-directed movements of 3 to 5-day-old neonates agreed with the two-thirds power law. Thus our data suggest an empirical hypothesis with potentially important implications for early diagnosis: atypical kinematics in individuals that go on to develop autism might be present from birth.

In conclusion, horizontal sinusoidal arm movements generated by adults with autism differed from those produced by typical control participants on measures of jerk, acceleration and velocity. Movement atypicalities in the autism group were correlated with a bias towards perceiving ‘unnatural’ motion on a biological motion categorization task and with the severity of autism. Given the importance of movement kinematics in one’s own navigation of the world and in the interpretation of others’ actions, movement kinematics might be an interesting avenue for further research and for therapeutic developments.

## Funding

This work was supported by a 4 year Wellcome Trust studentship award to J.C. (grant number 082910/Z/07/Z). S.J.B. is funded by a Royal Society University Research Fellowship and J.C. is currently funded by an AXA research fund award. The authors declare no conflicts of interest.

## Supplementary material

Supplementary material is available at *Brain* online.

Supplementary Data

## Abbreviations

ADOS: Autism Diagnostic Observation Schedule
CNS: central nervous system
SEM: standard error of the mean
UCL: University College London
