# Supplementary material for: Atypical basic movement kinematics in autism spectrum conditions
Source: Brain. 2013 Aug 26;136(9):2816–24. doi: 10.1093/brain/awt208 (PMC4017873; doi:10.1093/brain/awt208)
Supplement: Supplementary Data [file supp_136_9_2816__index.html]

Supplementary Data 

# Atypical basic movement kinematics in autism spectrum conditions

## Supplementary Data

files

**Files in this Data Supplement:**

- Supplementary Data - docx file
