# Supplementary material for: Atypical basic movement kinematics in autism spectrum conditions
Source: Brain. 2013 Aug 26;136(9):2816–24. doi: 10.1093/brain/awt208 (PMC4017873; doi:10.1093/brain/awt208)
Supplement: Supplementary Data [file supp_awt208_brain-2013-00530-File006.docx]

SUPPLEMENTARY MATERIALS

| Kinematic Feature | Effect | Statistics |
| --- | --- | --- |
| Velocity | Group main effect | F(1,27) = 7.888, p = 0.009, η_p_^2^  = 0.226 |
|  | Timepoint main effect | F(1,27) = 158.268, p <0.001, η_p_^2^  = 0.854 |
|  | Group x timepoint interaction | F(1,27) = 7.979, p = 0.009, η_p_^2^  =0.228 |
| Acceleration | Group main effect | F(1,27) = 8.806, p = 0.006, η_p_^2^  = 0.246 |
|  | Timepoint main effect | F(1,27) = 178.013, p <0.001, η_p_^2^  = 0.868 |
| Jerk | Group main effect | F(1,27) = 6.265, p = 0.019, η_p_^2^  = 0.1888 |

**S1: Supplemental Table.** Significant main effects and interactions for 2x2 ANOVAs with between-subjects factor group (autism vs control) and within-subjects factor timepoint (end vs middle). All other main effects and interactions were non-significant (p > 0.05).

**S2: Definitions of natural employed in the biological motion categorisation task**

In the Biological condition the participant was told … “to consider ‘natural’ to mean that the hand moves in a way that a person would typically move if asked to make an up and down movement with their arm. This person should not be thought to be in a particular emotional state such as angry or happy. An ‘unnatural’ movement would be one in which the hand moves in an odd, unusual way”… .

For the Non-biological condition the participant should: “consider ‘natural’ to mean that the ball moves in a way that would typically be seen if it were dropped (not thrown) from a point just above the computer screen [this point was demonstrated by the experimenter]. An ‘unnatural’ movement would be one in which the ball falls in an odd, unusual way”....


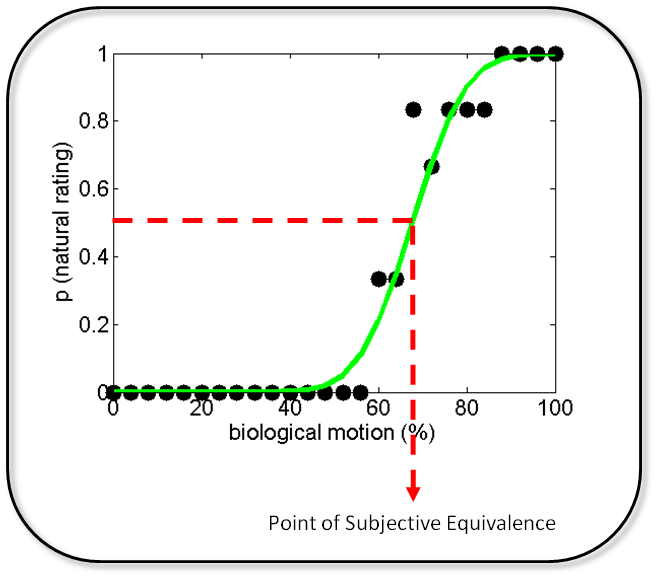


**S3: Example psychometric function.** Data were modelled by fitting cumulative Gaussians to estimate psychometric functions. Separate functions for biological and non-biological tasks were modelled for each participant, and the point of subjective equivalence (PSE) was estimated. The PSE denotes the ratio of ‘signal’ (MJ or G) to noise (CV) at the point where participants are equally likely to judge a stimulus as natural or unnatural. Thus, a high PSE indicates that, despite the stimulus comprising a high ratio of signal to noise, it is still judged as natural only 50% of the time; thus demonstrating a bias towards unnatural judgements. The above graph shows a single participant’s probability of natural judgements plotted against the objective percentage of minimum-jerk biological motion (signal) present in the stimulus. The green line indicates the psychometric function and the red dotted line illustrates calculation of the PSE.
